# Phenotypic Integration Facilitates Plasticity Through Reorganization of Traits Under Multiple Stress: Evidence From Asexual Water Flea Genotypes

**DOI:** 10.1002/ece3.74304

**Published:** 2026-09-10

**Authors:** Xiaofei Tian, Wenping Feng, Xiumei Zhang

**Affiliations:** ^1^ Fishery College Zhejiang Ocean University Zhoushan China

**Keywords:** *Daphnia pulex*, differential plasticity, genetic distance, multiple stress, phenotypic integration, phenotypic plasticity

## Abstract

Traditional theory posits that phenotypic integration—the structured covariation among traits—constrains phenotypic plasticity, thereby limiting adaptive responses. In contrast, emerging perspectives suggest integration may instead facilitate plasticity through coordinated trait adjustments. The mechanistic basis of this relationship, particularly how differential plasticity among traits shapes integration, remains empirically unresolved. To test these competing hypotheses, we exposed five genotypes of an asexual *Daphnia* cf. *pulex* to a factorial design of six temperature × food environments. We measured key functional traits (activities of five digestive enzymes and body length) and quantified phenotypic integration, trait plasticity, and differential plasticity. We then assessed: (i) how genotype and environment shape integration structure, (ii) the relationship between integration strength and plasticity magnitude, and (iii) how integration shapes correlated versus differential plasticity across environments. Our results show that phenotypic integration is itself highly plastic, dynamically reconfigured by genotype‐by‐environment interactions. The integration–plasticity relationship was context‐dependent: under low‐food conditions, stronger integration was associated with greater plasticity, supporting a facilitative rather than constraining role in this system. Notably, the stability of trait correlations across environments declined significantly as the magnitude of differential plasticity between traits increased. This identifies plasticity coordination as the mechanistic link through which integration buffers organisms against environmental variation. Variation in integration and plasticity was independent of pairwise genetic distance among clones. Our findings challenge the classic constraint model by demonstrating that phenotypic integration is a labile property that facilitates functional plasticity through coordinated trait adjustments. Specifically, variation in differential plasticity serves as a critical mechanistic determinant of this process. This capacity for context‐dependent reorganization of trait relationships is largely decoupled from genetic constraints in our study system. It positions integration as a potential dynamic facilitator of rapid adaptation, potentially underpinning the success of asexual lineages in fluctuating environments.

## Graphical abstract

This study demonstrates that phenotypic integration, the coordinated covariation among traits, is itself a plastic and context‐dependent property in clonal *Daphnia*. Contrary to the classic constraint model, we found that stronger integration can facilitate greater plasticity, particularly under low‐food conditions, with differential plasticity between traits serving as the key mechanism for dynamically rewiring trait correlations. This capacity for environmental reorganization of phenotypic architecture, largely independent of genetic background, positions integration as a dynamic facilitator of rapid adaptation in asexual lineages.

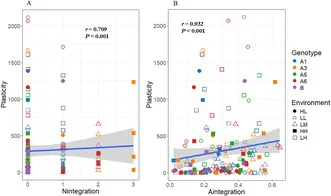

## 1 Introduction

Phenotypic plasticity, the ability of a single genotype to produce varying phenotypes in response to different environmental conditions, is widely acknowledged as a crucial adaptive mechanism for responding to environmental change (Pigliucci 2001; West‐Eberhard 2003; Murren et al. 2005; Richards et al. 2006; Vinton et al. 2022). This capacity is particularly relevant under global climate change, where rising temperatures and resource fluctuations create complex multi‐stressor environments that challenge organismal adaptation (Zhang et al. 2024). Understanding how organisms balance immediate phenotypic responses with long‐term evolutionary adaptation represents a fundamental challenge in evolutionary ecology (West‐Eberhard 2003; Price et al. 2003; Ghalambor et al. 2007).

Critically, the expression and magnitude of plasticity are intrinsically linked to phenotypic integration—the structured covariation among traits within an organism (Pigliucci and Preston 2003). Integration forms functional modules that reflect the correlation among traits (Gianoli and Palacio‐López 2009). Moreover, phenotypic integration is not a static property but is itself environmentally contingent, as abiotic stress can alter trait correlations, suggesting it is a labile component of the phenotype (Gianoli 2004; Seguí et al. 2018; Benavides et al. 2021; Stotz and Gianoli 2026). This context‐dependency places phenotypic integration at the heart of a fundamental paradox. Traditional models posit that integration can constrain plasticity by coupling traits into rigid modules, reducing their independent responsiveness (Schlichting 1989; Gianoli and Palacio‐López 2009). Conversely, emerging evidence suggests integration may facilitate plasticity through coordinated trait adjustments (Matesanz et al. 2021; Stotz et al. 2025). The mechanistic link likely involves differential plasticity (divergent responses among traits), which can either maintain or reconfigure integration across environments (Schlichting 1986; Matesanz et al. 2021). This unresolved tension between constraint and facilitation underscores a key gap in understanding how organisms balance trait coordination with functional responsiveness under multiple environments.

Resolving this requires bridging phenotypic and genetic perspectives, because the relationship between integration and plasticity may be mediated by underlying genetic architecture (Pigliucci and Preston 2003; Colbourne et al. 2011; Kovuri et al. 2023). For instance, if integration patterns are genetically constrained, they may limit plasticity. But if they are environmentally sensitive and vary predictably across gradients (Zimmermann et al. 2016; Matesanz et al. 2021), they may facilitate plasticity through rapid reorganization of trait relationships and enhance adaptive capacity. Distinguishing between these mechanisms requires examining how genetic divergence and phenotypic architecture interact across environmental gradients. Specifically, we test whether integration patterns are conserved across genotypes or shift predictably along environmental axes.

Here, we test these competing predictions using the asexual *Daphnia* cf. *pulex* system, which is particularly well‐suited for examining the relationship between phenotypic integration and plasticity. The clonal reproduction of *Daphnia* allows us to isolate phenotypic responses from genetic confounding, as genetically identical individuals can be exposed to different environments. Moreover, *Daphnia* exhibits well‐documented plasticity in life‐history and physiological traits in response to temperature and food availability, providing a rich context for studying integration–plasticity dynamics (Tian et al. 2019, 2022, 2024). The JPN1 lineage, which successfully colonized Japanese lakes from its native North American range despite obligatory parthenogenesis (So et al. 2015), offers a unique opportunity to examine how integration and plasticity facilitate adaptation in novel environments. High covariation among JPN1 genotypes suggests that this lineage has evolved functional integration that supports adaptability, potentially compensating for limited genetic recombination (Tian et al. 2019, 2024). Collectively, these features make the *Daphnia* cf. *pulex* system particularly suitable for disentangling the relationship between integration and plasticity under multifactorial environmental conditions.

In this study, we focus on digestive enzyme activity because they operate at the critical interface of energy acquisition and allocation, directly mediating the temperature‐food nexus (Wojewodzic et al. 2011; Thunell et al. 2022). Enzyme activity reflects how organisms balance metabolic demands across environments: thermal variation drives shifts in kinetic requirements, while food availability governs substrate supply and stoichiometric investment (Wagner and Frost 2012; Schwarzenberger and Fink 2018; Tian et al. 2019, 2023, 2024). This dual responsiveness makes digestive enzymes ideal indicators of how organisms resolve the trade‐off between energy demands and supplies. Using the *Daphnia* model system under multifactorial environments (temperature × food regimes), we address four core questions: (1) Do organisms achieve environment‐specific integration peaks rather than fixed architectures? (2) Does integration constrain or facilitate plasticity under combined stressors? (3) How does integration shape trait coordination versus differential plasticity among traits? (4) Are integration and plasticity patterns phylogenetically constrained or environmentally determined? Our results challenge traditional constraint models and offer a new perspective on integration as a dynamic facilitator of phenotypic adaptation, with implications for evolutionary ecology and conservation biology.

## 2 Methods

### 2.1 Experimental Materials

The study utilized five clonal lines of *Daphnia* cf. *pulex* (JPN1 lineage), originally isolated from Japanese ponds and small lakes (see Table S1 for details) and previously characterized for their life‐history traits, intraspecific competitive ability, phenotypic plasticity, and digestive enzyme activity profiles by Tian et al. (2019, 2022, 2024). These genotypes were maintained under long‐term laboratory culture conditions. Organisms were fed the green alga *Scenedesmus obliquus* every 3 days at a concentration of 2 mg C L^−1^. The alga was cultured axenically using COMBO medium, a defined freshwater culture medium for algae and zooplankton (Kilham et al. 1998), within a continuous flow‐through system that maintained a 0.5 L daily dilution rate.

For each genotype, a single healthy adult female was randomly selected from long‐term stock cultures to serve as the founding mother. All experimental individuals originated from this single mother. Experimental neonates were obtained from the 3rd brood of females from the second or third generations, and only individuals less than 24 h old were selected for use. Prior to experimentation, they were acclimated in 900 mL bottles filled with 600 mL of COMBO medium, to which *S. obliquus* was added at a concentration of 2.0 mg C L^−1^. Acclimation occurred in a controlled environment room set to 20°C with a 14:10 h light: dark photoperiod. Daphnids were fed daily, and the medium was refreshed every other day. To prevent crowding effects, population density was carefully regulated and never exceeded 30 individuals per 600 mL (i.e., 1 individual per 20 mL of medium) across all clonal lines. Experiments were conducted under six treatments: 3 temperatures (16°C, 20°C, 24°C) × 2 food levels (0.2, 2.0 mg C L^−1^). Selected temperatures (16°C, 20°C, 24°C) span the typical summer surface water range in northern temperate lakes where *Daphnia* occurs (Makino et al. 2011; So et al. 2015; Maruoka and Urabe 2020). The food concentrations of 0.2 and 2.0 mg C L^−1^ represent limiting and saturating food conditions, respectively, covering the natural range of food availability experienced by *Daphnia* in temperate lakes (Tian et al. 2019). For each genotype, five neonates were assigned to each of the six treatments, with each neonate placed individually in a separate 50 mL bottle (i.e., five biological replicates per treatment per genotype, totaling 30 individuals per genotype across all treatments). During the 5‐day experimental period, the medium was renewed every 2 days and *Daphnia* were fed daily. At the end of the 5‐day period, five individuals per treatment group were rinsed twice with distilled water and processed for enzyme activity assays and body size measurement.

### 2.2 Assay of Digestive Enzyme Activity and Body Sizes

Procedures for enzyme activity and body size measurements followed the protocol established by Tian et al. (2019). For measurements of body length, these individuals were placed under a microscope where media was removed until the animals were properly positioned. Using an Olympus DP20 camera (Olympus, Tokyo, Japan) mounted on an Olympus SZH10 stereomicroscope (Olympus, Tokyo, Japan), images of *Daphnia* were captured at a magnification of 40×, and their body length from the base of the tail‐spine to the top of the head was subsequently measured using ImageJ software (National Institutes of Health, Bethesda, USA). The same individuals were then used for the assay of digestive enzyme activity.

Enzyme activities for five key digestive enzymes were quantified fluorometrically using specific 4‐methylumbelliferyl or coumarin‐based substrates: 4‐methylumbelliferyl beta‐D‐glucoside for beta‐glucosidase (EC 3.2.1.21), 4‐methylumbelliferyl butyrate for lipase (EC 3.1.1.3 and related), L‐arginine‐7‐amido‐4‐methylcoumarin hydrochloride for arginine aminopeptidase (EC 3.4.11.6), L‐alanine‐4‐methyl‐7‐coumarinylamide trifluoroacetate for alanine aminopeptidase (EC 3.4.11.2), and 4‐methylumbelliferyl phosphate for alkaline phosphatase (EC 3.1.3.1), following the protocol detailed in our previous studies (Tian et al. 2019, 2024). The protein content of the supernatant was determined using the bicinchoninic acid (BCA) assay (Smith et al. 1985) to normalize enzyme activity data.

### 2.3 Metrics of Phenotypic Integration and Plasticity

#### 2.3.1 Phenotypic Integration

To quantify phenotypic integration, we first constructed pairwise trait correlation matrices for each genotype under each experimental condition (Figure S1). All correlations are based on strictly matched, individual‐level data. As described in the preceding section, for each individual used in enzyme assays, body length was measured immediately prior to homogenization. Therefore, all traits (body length and the five digestive enzyme activities: beta‐glucosidase, lipase, alkaline phosphatase, arginine aminopeptidase, and alanine aminopeptidase) for correlation analysis were obtained from the same individuals, ensuring perfect per‐individual pairing of all data points. Pairwise associations among traits were quantified using Pearson correlation coefficients. Following established methods (Pigliucci and Marlow 2001; Murren et al. 2002; Gianoli 2004; Gianoli and Palacio‐López 2009), we quantified phenotypic integration using two complementary metrics. First, Nintegration is defined as the number of statistically significant correlations a given trait has with all other measured traits, capturing the connectedness of a trait within the correlation network. Although Nintegration is the more commonly adopted measure, its value is inherently sensitive to sample size and statistical power, as the detection of significant correlations depends on the number of available pairwise comparisons. Second, Aintegration is defined as the average squared Pearson correlation coefficient of a given trait with all other measured traits, providing a magnitude‐based measure of integration strength (Pigliucci et al. 1991). As discussed in Pigliucci et al. (1991), unlike Nintegration which depends on significance testing and is sensitive to sample size, Aintegration offers a more stable estimate of integration magnitude under small‐sample conditions. Both Nintegration and Aintegration represent trait‐level integration indices, capturing per‐trait connectedness within the correlation matrix. To evaluate the statistical power of the pairwise Pearson correlations underlying these integration indices given the limited sample size (*n* = 5 per group), we conducted a post hoc power analysis using the ‘pwr’ package in R (Champely 2020). Details are provided in Supporting Information (Figure S2).

#### 2.3.2 Phenotypic Plasticity

To standardize comparisons of environmental responsiveness across traits and genotypes, and given that no single environment serves universally as a reference, we adopted a genotype‐specific plasticity metric based on deviation from a common “reference” condition. Since the 2.0 mg C L^−1^—20°C regime represents a long‐term (> 10 years in laboratory culture) stable and favorable environment for these populations, we defined it as the baseline. For each genotype (*g*) and trait (*i*), phenotypic plasticity in any other environment (*e*) was calculated as the absolute difference between the mean phenotype in e and the mean of that same genotype and trait in the reference condition:

(1) $${\text{Plasticity}}_{\mathrm{ige}}=|x_{\mathrm{ige}}-x_{\mathrm{ig},\mathrm{ref}}|$$

where *x*_*ige*_ is the mean value of trait *i* for genotype *g* in environment *e*, *x*_*ig*,ref_ is the mean of trait *i* for genotype *g* in the reference environment (2.0 mg C L^−1^, 20°C). This approach directly quantifies the magnitude of phenotypic shift from the favorable baseline, facilitating interpretation of plasticity in ecologically relevant terms.

### 2.4 Statistical Analysis

#### 2.4.1 Effect of Genotype and Environment on Phenotypic Integration

To evaluate the effects of genotype and environment on trait integration, correlation matrices were systematically compared both among genotypes within each environment and among environments within each genotype using three approaches. First, to test for differences in correlation matrices, we conducted comprehensive pairwise equality tests. For each of the six environments, we performed all possible pairwise comparisons among the correlation matrices of the five genotypes (10 comparisons per environment). Conversely, for each of the five genotypes, we compared its correlation matrix across all pairs of the six environments (15 comparisons per genotype). Each pairwise comparison was executed using the *cortest.normal* function (R package ‘psych’) (Revelle 2015), which tests the null hypothesis that two correlation matrices are identical and generates a chi‐square statistic. Second, considering that unequal matrices may still share similarities, a Common Principal Components (CPCs) analysis was implemented following Flury's hierarchical hypothesis framework (Phillips and Arnold 1999; Roff et al. 2012). This method permits a more complex comparison of correlation architectures by testing a hierarchy of models, ranging from complete equality and proportionality to partial eigenvector sharing and full structural dissimilarity. Model selection within the CPC framework was guided by the Akaike Information Criterion (AIC), with models differing by less than 2 AIC units considered to have equivalent empirical support (Burnham and Anderson 2003). All CPC computations were executed using the ‘CPC’ R package (Pepler 2019). Finally, we estimated overall phenotypic integration using the variance of eigenvalues (*Var*) derived from pairwise trait correlation matrices, which serves as a widely used matrix‐level index of integration (INT), where high variance reflects high integration (Pavlicev et al. 2009; Damián et al. 2020). The variance for each genotype (*g*) in each environment (*e*) was estimated as follows:

(2) $${\mathrm{Var}}_{\mathrm{ge}}=\frac{\sum_{i}^{N}{\left(\lambda_{i}-1\right)}^{2}}{N}$$

where *N* is the trait number, *λ*_*i*_ is the eigenvalue of the *i*th principal component.

#### 2.4.2 Relationships Between Phenotypic Integration and Plasticity

We conducted three sets of analyses to examine the relationship between phenotypic integration and plasticity, as detailed below.

Step 1: Effect of integration on plasticity. To explore the effect of each integration metric (Nintegration and Aintegration) on phenotypic plasticity (PP), we performed a Generalized Linear Mixed Model (GLMM) analysis. The phenotypic plasticity (PP) for each trait was calculated as the absolute deviation from the reference condition and subsequently log_10_(PP + 1)‐transformed to normalize its distribution. The model was specified as follows:

(3) $$\mathrm{PP}\sim \left(1|\;\text{Genotype}\right)+\text{Environment}+\text{Integration}$$

where Integration represents either Nintegration or Aintegration in separate models. If the regression coefficient of integration was significant, we concluded that the integration could affect the phenotypic plasticity. The conventional coefficient of determination (*r* ^2^) was used to quantify the percentage of phenotypic variation explained.

Step 2: Quantifying differential plasticity between trait pairs. Following Matesanz et al. (2021), we tested for differential plasticity (non‐parallel reaction norms) between each pair of traits. Raw phenotypic values were log_10_‐transformed and structured into a single response vector (TV) with a factor column Trait indicating trait identity (A or B). We then fitted a separate linear mixed model for each trait pair using the *lmer* function (R package ‘lme4’) (Bates et al. 2015). The model structure was:

(4) $$\mathrm{TV}\sim \text{Trait}+\text{Environment}+\text{Trait}:\text{Environment}+\left(1|\text{Genotype}\right)$$

A significant Trait × Environment interaction is interpreted as differential plasticity between the two traits, as evidenced by their non‐parallel reaction norms (Matesanz et al. 2021). (1|Genotype) is a random intercept term, accounting for the non‐independence of measurements coming from the same clonal genotype across different environments and traits. Its significance was assessed via likelihood ratio test (LRT) comparing the full model against a reduced model without the random term. A significant LRT result (*p* < 0.05) indicates that genotype identity explains a significant portion of the variance in trait values, implying that the plastic responses of the two traits vary among genotypes.

Step 3: Linking differential plasticity to integration stability. We then examined if the degree of integration (i.e., correlation) between two traits across environments predicts the similarity in their plasticity. Specifically, we hypothesized that correlated traits would also co‐vary in their plastic responses. To do so, we first quantified the difference in plasticity between each pair of traits, as differential plasticity is inherently a pairwise comparison of reaction norm slopes (Matesanz et al. 2021). Following their approach, we focused on the magnitude, rather than the direction, of differences in plastic responses. We therefore calculated the absolute difference in plasticity (*Dpg*) for each genotype between two traits across each pair of environments as follows:

(5) $${\mathrm{Dpg}}_{\mathrm{xy}}=\frac{\left\|m_{\mathrm{ix}}-m_{\mathrm{iy}}|-|m_{\mathrm{jx}}-m_{\mathrm{jy}}\right\|}{|m_{\mathrm{ix}}-m_{\mathrm{iy}}|+|m_{\mathrm{jx}}-m_{\mathrm{jy}}|}$$

where *m*_*ix*_ and *m*_*iy*_ are the mean values of trait *i* in environments *x* and *y*, respectively, and *m*_*jx*_ and *m*_*jy*_ are the corresponding mean values of trait *j*. This metric provides a standardized, unsigned measure of dissimilarity in trait plasticity across environments, ranging from 0 (identical magnitude) to 1 (maximally divergent magnitude). A larger *Dpg* value indicates a greater divergence in the magnitude of plastic responses between the traits, irrespective of whether both traits increase, decrease, or change in opposite directions across environments (Matesanz et al. 2021). A worked example of this calculation is provided in Supporting Information. Then, for each genotype, we quantified changes in phenotypic integration between environments by computing the matrix of differences in pairwise trait correlations across each pair of environments. Specifically, differences (or similarities) in pairwise trait integration (*Dig*) were assigned a ternary code: 1 for traits significantly correlated in both environments, 0 for correlation in only one environment, and −1 for no significant correlation in either environment (Matesanz et al. 2021). Finally, to evaluate the relation between divergent trait plasticity and the maintenance of trait correlations, we conducted a GLMM analysis. The model was developed as:

(6) $$\mathrm{Dpg}\sim \mathrm{Dig}+\left(1|\text{Genotype}\right)$$

A negative and significant coefficient for plasticity difference is interpreted as evidence that traits with more dissimilar plasticity are more likely to alter their correlation across environments.

#### 2.4.3 Relationships Between Genetic Differences and Phenotypic Differences in Plasticity and Integration

Genetic dissimilarity data were collected from Tian et al. (2019), based on the uncorrected p‐distance (proportion of nucleotide differences) calculated from 135,933,993 aligned base pairs, and were used in this study to represent genetic dissimilarity. To clarify the relationship between genetic differences and phenotypic differences in integration and plasticity, we considered three phenotypic metrics: Nintegration, Aintegration, and phenotypic plasticity (PP). For each genotype, we estimated the mean value of Nintegration, Aintegration, and plasticity across environments. We then calculated the absolute differences in each of these three phenotypic metrics between each pair of genotypes using the genotype‐level mean values. Subsequently, Mantel tests were performed to evaluate the correlations between pairwise genetic distances and each of the three phenotypic difference matrices (Nintegration, Aintegration, and plasticity).

All statistical computations were conducted in R 4.4.1 (R Core Team 2024). Corresponding regression plots, including 95% confidence intervals, were employed via the *geom_smooth*() function in the ‘ggplot2’ package (Wickham 2016).

## 3 Results

### 3.1 Relationship Between Phenotypic Integration and Phenotypic Plasticity

#### 3.1.1 Integration Metrics and Plasticity Magnitude

**FIGURE 1:**
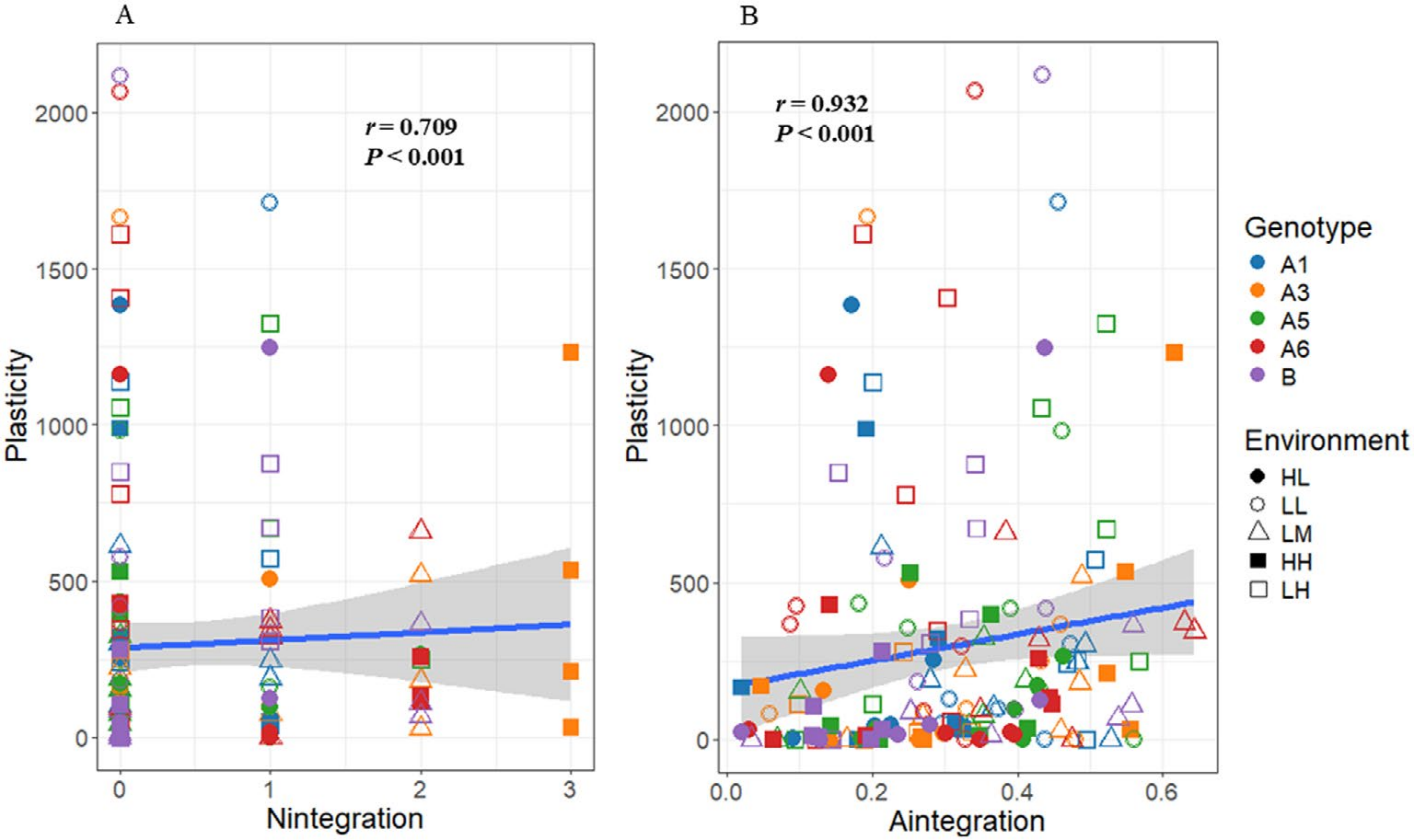
Phenotypic plasticity of *Daphnia* cf. *pulex* genotypes fitted against Nintegration (A) and Aintegration (B). Nintegration means number of statistically significant correlations a trait has with all other measured traits. Aintegration means average squared Pearson correlation coefficient of a trait with all other measured traits. Treatment codes are defined as follows: HL, high food (2.0 mg C L^−1^) × low temperature (16°C); LL, low food (0.2 mg C L^−1^) × low temperature (16°C); HM, high food (2.0 mg C L^−1^) × moderate temperature (20°C); LM, low food (0.2 mg C L^−1^) × moderate temperature (20°C); HH, high food (2.0 mg C L^−1^) × high temperature (24°C); LH, low food (0.2 mg C L^−1^) × high temperature (24°C). Correlation coefficients (*r*) were calculated from transformed data (log_10_(PP + 1)‐transformed plasticity and integration indices). Scatter points and regression lines are based on raw (untransformed) data at the individual level.

We quantified phenotypic integration for each trait via two complementary metrics: Nintegrition, which counted its number of significant correlations, and Aintegrition, which represented the average of its squared correlation coefficients. Our GLMM analysis revealed that both integration metrics (Nintegrition and Aintegrition), significantly and positively influenced phenotypic plasticity (Table S2). Aintegration was a stronger predictor, explaining approximately 86% of the variation in plasticity, and this relationship was robust across all environmental conditions (Figures 1B and S3). In contrast, Nintegration explained approximately 50% of the variation, and its effect was strongly modulated by the environment: the association was positive under most conditions but became negative under combined low‐food and low‐temperature stress (Figures 1A and S3), indicating that a highly connected trait network could constrain plasticity under the most severe energy limitation. The role of genetic variation also differed between models. In the Nintegration model, the random effect of genotype was significant (LRT, *p* = 0.017, Table S2), indicating residual genetic differences not captured by network connectivity. Conversely, in the Aintegration model, the genotype effect was nonsignificant (*p* = 0.403, Table S2), suggesting that the average integration metric fully subsumed the genetically based variation in plasticity.

#### 3.1.2 Integration Stability and Differential Plasticity

**FIGURE 2:**
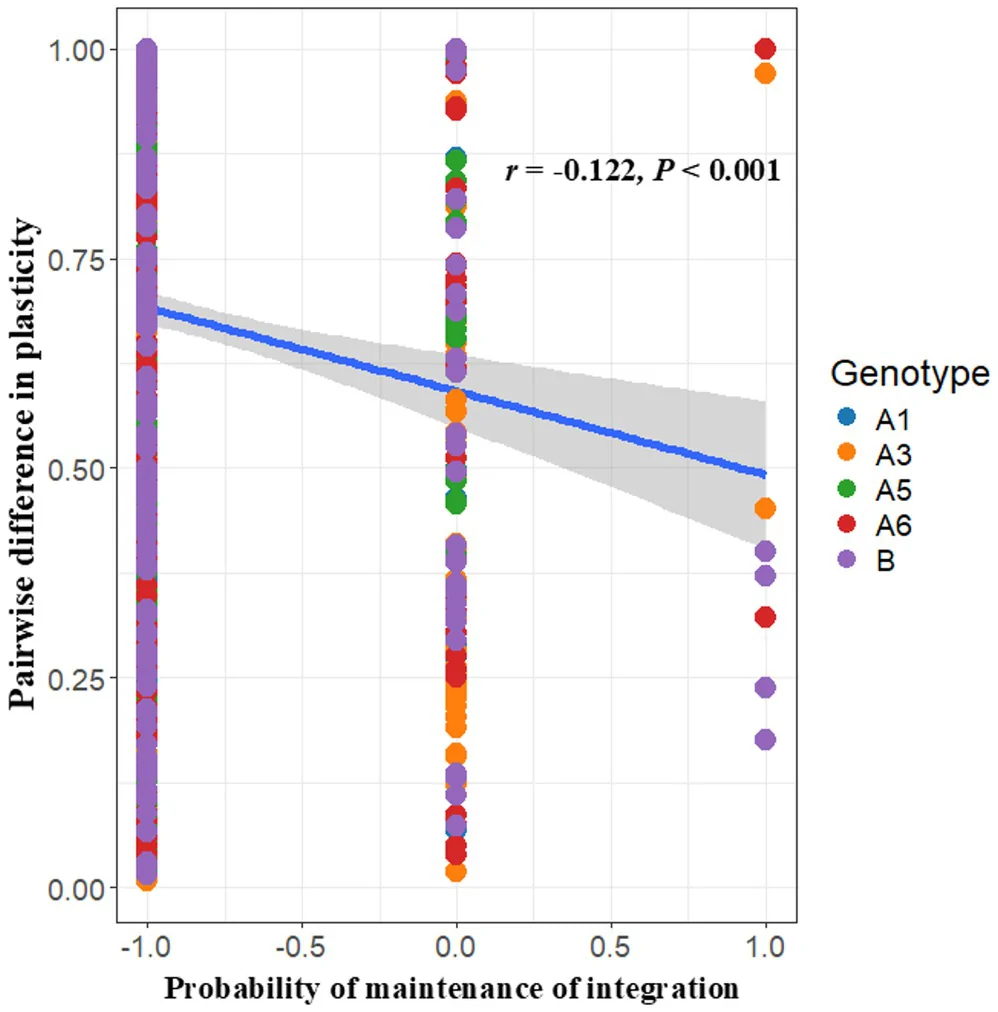
Relationship between the differences in phenotypic plasticity and the change in phenotypic integration in *Daphnia* cf. *pulex* genotypes.

Our analysis focused on how stability of trait correlations across environments affects the differences in trait plasticity. Model results showed a significant negative relationship: traits with greater instability in integration across environments exhibited greater differences in plasticity (i.e., those showing nonparallel norms of reaction and significant Trait × Environment interactions), a pattern that held across varying levels of initial trait correlation. Conversely, traits exhibiting correlated plasticity (parallel norms) tended to respond similarly across environments, maintaining stable integration (Figure 2). The random effect of genotype was negligible, indicating that this negative relationship was consistent across all genotypes. However, the model explained only 1.5% of the variance in plasticity differences (Table S3), suggesting that majority of variation in differential plasticity is driven by other factors, such as trait identity, environmental context, and genotype‐specific responses.

Among the 15 trait pairs, all but one (beta‐glucosidase vs. arginine amino‐peptidase) exhibited significantly differential plasticity (Table 1, Figure S4). Notably, the random effect of genotype was significant, suggesting that genotypes responded plastically in different ways for the two traits. Specifically, all genotypes showed similar plastic responses for 10 trait pairs (including beta‐glucosidase vs. lipase, beta‐glucosidase vs. alkaline phosphatase, lipase vs. arginine amino‐peptidase, and others) (Table 1, Figure S4). However, for the remaining five trait pairs, plasticity responses varied significantly among genotypes. Among these five pairs of traits, two trait pairs were between body size and some of the five enzyme activities (i.e., lipase vs. body length, alanine amino‐peptidase vs. body length) (Table 1, Figure S4).

**TABLE 1:** Results of linear mixed models examining sources of variation in raw trait values for each of the 15 trait pairs.

Trait comparison	T × E		Genotype		Trait pair		Environment	
	*F*	*p*	*F*	*p*	*F*	*p*	*F*	*p*
Beta‐glucosidase vs. lipase	23.9	**< 0.001**	1.72	0.189	1314.99	**< 0.001**	35.54	**< 0.001**
Beta‐glucosidase vs. alkaline phosphatase	15.4	**< 0.001**	0.45	0.502	91.54	**< 0.001**	33.70	**< 0.001**
Beta‐glucosidase vs. arginine amino‐peptidase	1.44	0.210	1.34	0.247	35.37	**< 0.001**	62.49	**< 0.001**
Beta‐glucosidase vs. alanine amino‐peptidase	3.29	**0.006**	4.03	**0.045**	78.40	**< 0.001**	45.18	**< 0.001**
Beta‐glucosidase vs. body length at day five	28.73	**< 0.001**	0.12	0.726	301.93	**< 0.001**	28.75	**< 0.001**
Lipase vs. alkaline phosphatase	11.54	**< 0.001**	4.09	**0.043**	11,678.28	**< 0.001**	29.54	**< 0.001**
Lipase vs. arginine amino‐peptidase	25.27	**< 0.001**	0.07	0.791	2001.75	**< 0.001**	44.76	**< 0.001**
Lipase vs. alanine amino‐peptidase	15.62	**< 0.001**	0.04	0.838	3114.82	**< 0.001**	25.04	**< 0.001**
Lipase vs. body length at day five	50.97	**< 0.001**	7.22	**0.007**	101,717.57	**< 0.001**	19.39	**< 0.001**
Alkaline phosphatase vs. arginine amino‐peptidase	16.31	**< 0.001**	2.47	0.116	280.59	**< 0.001**	42.14	**< 0.001**
Alkaline phosphatase vs. alanine amino‐peptidase	8.75	**< 0.001**	3.23	0.072	471.59	**< 0.001**	24.81	**< 0.001**
Alkaline phosphatase vs. body length at day five	27.59	**< 0.001**	2.32	0.128	27,596.23	**< 0.001**	8.58	**< 0.001**
Arginine amino‐peptidase vs. alanine amino‐peptidase	5.08	**< 0.001**	9.36	**0.002**	5.69	**0.01**	52.80	**< 0.001**
Arginine amino‐peptidase vs. body length at day five	35.50	**< 0.001**	1.07	0.299	10,736.80	**< 0.001**	34.93	**< 0.001**
Alanine amino‐peptidase vs. body length at day five	17.03	**< 0.001**	11.47	**< 0.001**	15,651.03	**< 0.001**	18.85	**< 0.001**

*Note:* Models included fixed effects of Trait (identity of the two traits compared), Environment (six temperature × food treatments), and their interaction (Trait × Environment (T × E)) with Genotype fitted as a random intercept. The significance levels are shown in bold. The main effect of Environment tests for overall phenotypic differences among the six treatments. Trait pair represents the two traits being compared in each linear mixed model (e.g., body length and lipase; body length and alkaline phosphatase), indicating which phenotypic attributes are involved in the differential plasticity analysis.

### 3.2 Effect of Genotype and Environment on Phenotypic Integration

Both genotype and environment significantly affected the structure of the correlation matrices. Pairwise equality tests revealed significant differences both across environments within each genotype and across genotypes within each environment (Table S4), indicating that phenotypic integration is jointly shaped by genetic background and environmental context. Flury's CPC analysis further confirmed that the correlation matrices differed across comparisons, with the best‐fitting model (Heterogeneity) indicating unrelated matrices both across environments within each genotype and across genotypes within each environment (Table 2). These results demonstrate that the entire correlation structure, not just individual trait pairs, undergoes environment‐ and genotype‐dependent reorganization.

**TABLE 2:** Flury common principal component (CPC) test for correlation matrix comparisons of seven functional traits measured among environments within each *Daphnia* cf. *pulex* genotype (A), among genotypes within each environment (B).

	Model based approach	AIC	#CPCs
	Model		
**(A) Genotype**			
A1	Equality	523.27	6
	Proportionality	531.83	6
	CPC	469.52	6
	CPC(4)	466.72	4
	CPC(3)	479.56	3
	CPC(2)	489.86	2
	CPC(1)	349.58	1
	**Heterogeneity**	**210.00**	**0**
A3	Equality	514.57	6
	Proportionality	521.96	6
	CPC	463.31	6
	CPC(4)	469.55	4
	CPC(3)	476.65	3
	CPC(2)	472.54	2
	CPC(1)	361.08	1
	**Heterogeneity**	**210.00**	**0**
A5	Equality	535.43	6
	Proportionality	542.79	6
	CPC	456.69	6
	CPC(4)	463.02	4
	CPC(3)	470.36	3
	CPC(2)	489.54	2
	CPC(1)	357.87	1
	**Heterogeneity**	**210.00**	**0**
A6	Equality	537.98	6
	Proportionality	545.08	6
	CPC	487.78	6
	CPC(4)	491.42	4
	CPC(3)	481.82	3
	CPC(2)	499.05	2
	CPC(1)	367.48	1
	**Heterogeneity**	**210.00**	**0**
B	Equality	520.31	6
	Proportionality	526.77	6
	CPC	459.24	6
	CPC(4)	466.43	4
	CPC(3)	433.58	3
	CPC(2)	436.31	2
	CPC(1)	364.35	1
	**Heterogeneity**	**210.00**	**0**
**(B) Environment**			
2.0 mg C L^−1^—16°C	Equality	426.84	6
	Proportionality	434.22	6
	CPC	336.65	6
	CPC(4)	342.22	4
	CPC(3)	348.83	3
	CPC(2)	348.07	2
	CPC(1)	272.53	1
	**Heterogeneity**	**168.00**	**0**
0.2 mg C L^−1^—16°C	Equality	424.34	6
	Proportionality	429.14	6
	CPC	339.48	6
	CPC(4)	344.78	4
	CPC(3)	357.62	3
	CPC(2)	345.33	2
	CPC(1)	291.22	1
	**Heterogeneity**	**168.00**	**0**
2.0 mg C L^−1^—20°C	Equality	427.3	6
	Proportionality	433.47	6
	CPC	404.81	6
	CPC(4)	403.49	4
	CPC(3)	404.59	3
	CPC(2)	358.44	2
	CPC(1)	302.19	1
	**Heterogeneity**	**168.00**	**0**
0.2 mg C L^−1^—20°C	Equality	447.99	6
	Proportionality	454.67	6
	CPC	379.01	6
	CPC(4)	383.98	4
	CPC(3)	392.54	3
	CPC(2)	382.06	2
	CPC(1)	277.43	1
	**Heterogeneity**	**168.00**	**0**
2.0 mg C L^−1^—24°C	Equality	424.95	6
	Proportionality	428.4	6
	CPC	363.81	6
	CPC(4)	364.82	4
	CPC(3)	358.23	3
	CPC(2)	345.7	2
	CPC(1)	260.64	1
	**Heterogeneity**	**168.00**	**0**
0.2 mg C L^−1^—24°C	Equality	429.27	6
	Proportionality	435.59	6
	CPC	356.81	6
	CPC(4)	359.04	4
	CPC(3)	369.78	3
	CPC(2)	357.92	2
	CPC(1)	286.21	1
	**Heterogeneity**	**168.00**	**0**

*Note:* Akaike information criterion (AIC) is shown for each model; the lowest AIC value gives the most likely model for the model‐building approach. #CPCs denotes the number of common principal components retained in each model. The hierarchical CPC models are: Equality means correlation matrices are equal (identical structure and magnitude); Proportionality means matrices share the same eigenvectors but differ by a scalar multiple (proportional scaling); Heterogeneity means matrices are unrelated, with all eigenvectors and eigenvalues differing (no shared structure). Intermediate models CPC(*k*) retain *k* common principal components (*k* = 4, 3, 2, 1), with the remaining components being group‐specific.

The magnitude of phenotypic integration, estimated by the INT index, also varied substantially among genotypes and environments (Table S5). Environments inducing the tightest integration differed by genotype: low food/low temperature for A1 and A5, low food/moderate temperature for A6 and B, and high food/high temperature for A3. In these cases, integration was characterized by a subset of robust, positive associations that formed the core of the integrated phenotype. Certain trait relationships were particularly stable across different genotypes (Figure S1). For example, lipase showed a positive correlation with body length under low food/low temperature in genotype A1. Alanine amino‐peptidase was positively related to body length under the same conditions in A5. And alkaline phosphatase showed positive correlations with body length under low food/moderate temperature in A6.

Given the limited sample size (*n* = 5 per group), we assessed the reliability of the integration metrics. Across all 30 groups, the mean absolute pairwise correlation (|*r*|) was 0.48 (SD = 0.12), with group means ranging from 0.36 to 0.72 (Table S6). These values are well below the range where statistical power becomes adequate at *n* = 5 (Figure S2). Notably, when correlations were statistically significant, they were typically very strong (*r* = 0.80–0.99, mostly > 0.90), corresponding to a power of approximately 0.84. This pattern suggests that Nintegration values are unlikely to overestimate trait connectedness. Rather, limited power may lead to underestimation of genuine correlations, making Nintegration a conservative estimate. Aintegration, which averages squared correlation coefficients without relying on significance thresholds, is not subject to the same power limitation and provides a complementary, more stable measure under these conditions.

### 3.3 Relationship Between Genetic Distance and Phenotypic Differences in Phenotypic Plasticity and Integration

**FIGURE 3:**
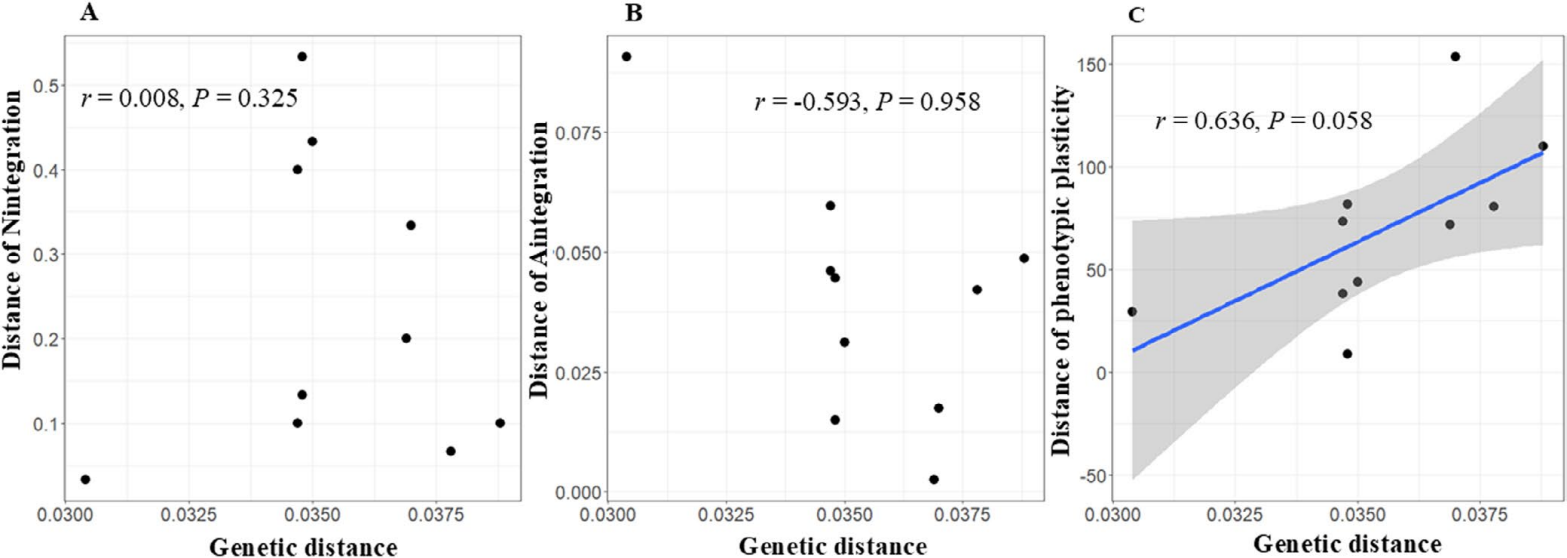
Relationship between genetic distance and phenotypic distance in Nintegration (A), Aintegration (B) and plasticity (C) between *Daphnia* cf. *pulex* genotypes. The correlation coefficient (*r*) and probability of significance (*p*) in the Mantel test are shown in each panel.

We tested whether genetic distance was associated with phenotypic divergence in plasticity and integration. Genetic distances were estimated from previously published phylogenetic relationships (Tian et al. 2019) and analyzed for correlation with phenotypic differences. The analysis revealed no significant relationships for any of the phenotypic traits examined (Figure 3). No significant associations were found for most traits, but plasticity divergence showed a marginally significant positive correlation with genetic distance (*r* = 0.636, *p* = 0.058).

## 4 Discussion

Our study demonstrates that phenotypic integration in *D*. cf. *pulex* is a highly dynamic and context‐dependent property, rather than a static constraint. Contrary to the traditional view that tight integration inherently limits phenotypic flexibility, we find that integration can both facilitate and constrain plasticity, depending on the specific metric of integration and the environmental context. This fundamental shift in perspective, from integration as a fixed architecture to integration as a labile, functional trait, forms the core of our findings and compels a rethinking of how trait coordination enables organisms to navigate complex, fluctuating environments. The following sections elaborate on the mechanisms, ecological implications, and evolutionary significance of this dynamic relationship.

### 4.1 The Association Between Phenotypic Integration and Plasticity

Our study reveals a significant positive association between phenotypic integration and plasticity, yet the nature of this relationship depends critically on how integration is quantified. While average integration strength (Aintegration) showed a robust, positive link with plasticity across all environments, the effect of network connectivity (Nintegration) was strongly modulated by the environment. Most critically, the Nintegration‐plasticity association shifted from positive under most conditions to negative under the combined stress of low food and low temperature. Notably, the extreme data points driving this pattern corresponded to specific environmental contexts rather than random outliers, further supporting the environment‐specific nature of the relationship. This reversal provides empirical support for the emerging view that the integration–plasticity link is not fixed but condition‐dependent (Zimmermann et al. 2016; Stotz et al. 2025). This nuanced, context‐dependent pattern extends and provides a mechanistic case study for the findings of a recent meta‐analysis, which concluded that the integration–plasticity relationship is conditional and that integration can limit variation in plasticity among traits (Stotz et al. 2025). Our study complements this by showing that within a species, the effect of integration (especially network connectivity, Nintegration) on the magnitude of plasticity is itself environmentally contingent, and can shift from facilitative to constraining under extreme stress.

Despite the potential cost under extreme stress, a prominent functional pattern emerged: for most genotypes, the tightest phenotypic integration was consistently achieved under low‐food conditions. This convergence suggests that under chronic energy limitation, the benefits of a highly coordinated phenotype, such as minimizing wasteful allocation and enforcing efficient resource use, likely outweigh the risks of constrained plasticity under the rarest, most severe stress combinations. We interpret this as a functional outcome that could enhance fitness under resource scarcity, consistent with the idea that integration can be a component of invasiveness and adaptation (Godoy et al. 2012). However, we emphasize that this interpretation remains hypothetical in the absence of direct fitness measurements. The observed pattern could equally reflect non‐adaptive physiological constraints exposed under stress, and distinguishing between these alternatives will require experimental manipulation of integration coupled with fitness assays. Nevertheless, the theoretical and empirical basis for the adaptive value of tight integration under resource scarcity is strong (Lynch 1977; Gliwicz 1990; Auer et al. 2020). Notably, genotypes reached this integrated state under different thermal regimes, illustrating a diversity of physiological solutions to the common challenge of low food. This pattern underscores resource availability as a key driver shaping integrated phenotypes, with genetic background fine‐tuning the precise environmental context for optimal integration.

Moreover, our study also reveals how phenotypic integration structures the correlated plasticity among traits. We found that the strength of a trait correlation in a given environment strongly predicts the degree to which the two traits exhibit correlated plastic responses to that environment. This positive association was most pronounced under low‐food conditions, reinforcing the stabilizing role of tight integration under energy scarcity. However, the maintenance of trait correlations across different environments was not guaranteed. The breakdown of correlation in new environments was often associated with differential plasticity—divergent environmental responses between the two traits (Schlichting 1986; Matesanz et al. 2021). Indeed, 14 out of 15 trait pairs exhibited significant differential plasticity, which frequently decoupled their phenotypic associations when conditions changed. This indicates that while integration promotes coordinated change, differences in plasticity among traits provide the mechanistic basis for the dynamic reorganization of the correlation structure itself.

Analysis at the genotypic level revealed two underlying patterns. Plastic responses for 10 trait pairs were highly consistent across genotypes, suggesting canalized, core functional modules. In contrast, the coordination of the remaining five trait pairs, which included key linkages between body size and digestive enzymes, varied significantly among genotypes, implying evolutionary lability. This indicates that the integration between energy acquisition (digestion) and allocation (growth) is a principal axis of evolvable flexibility in *D*. cf. *pulex*. The stability of the former group may be crucial for maintaining core physiological functions, while the lability of the latter allows for functional adjustment of resource allocation strategies in response to selection.

Building on these findings, we propose that the plasticity of integration, the capacity to dynamically reconfigure phenotypic architecture, is itself a critical, evolvable property. Our observation of distinct stable and labile trait modules mirrors the architecture of phenotypic integration found to be under direct selection in other systems, such as the leaves of *Turnera velutina* (Damián et al. 2020). In that study, the magnitude of phenotypic integration among functional leaf traits was itself positively correlated with plant fitness, providing direct evidence that natural selection can act on the covariance structure of traits, not just on trait means. This supports our proposal that in *D*. cf. *pulex*, the ability to maintain robust core physiological modules while flexibly reorganizing resource allocation linkages in response to the environment (e.g., low‐food stress) constitutes a potential functional strategy. The fact that the phenotypic structure upon which selection acts is not fixed but can be environmentally reshaped (Schlichting 1986, 1989; Agrawal and Stinchcombe 2009) expands the evolutionary framework. Future work incorporating fitness assays, as in Damián et al. (2020), is needed to directly test whether the specific integration patterns we observed are indeed under positive selection. Consequently, natural selection in heterogeneous environments may favor genotypes not only for optimal trait values but also for possessing “rules of coordination” that enable optimal reconfiguration of trait relationships, balancing immediate physiological robustness with long‐term functional responsiveness.

### 4.2 Context‐Dependent Reorganization of Phenotypic Integration by Genotype and Environment

The phenotypic integration structure in *D*. cf. *pulex* is not static but is dynamically reshaped by genotype‐by‐environment (G × E) interactions, as indicated by the Flury CPC analysis showing unrelated correlation matrices. This functional plasticity in trait coordination underscores that integration itself constitutes a labile, context‐dependent property, reflecting the organism's capacity to reorganize its internal architecture in response to specific genetic and environmental contexts (Brown et al. 2022; Jiang et al. 2025). This finding extends the well‐established principle from plant ecology that phenotypic integration is environmentally contingent, often increasing under abiotic stress such as resource limitation or aridity (Gianoli 2004; Seguí et al. 2018; Benavides et al. 2021; Stotz and Gianoli 2026). Critically, our study not only demonstrates that this principle applies to an aquatic invertebrate, but also identifies low food as a key environmental driver and reveals that the resulting integration pattern is fine‐tuned by genetic background. This capacity for context‐dependent reorganization likely facilitates the maintenance of functional coherence while permitting environment‐responsive flexibility under heterogeneous conditions (Pigliucci 2001; Murren et al. 2005).

The specific environmental conditions that elicited the tightest phenotypic integration (highest INT index) varied considerably among genotypes. While this pattern suggests divergent adaptive strategies for managing energy under stress, we caution that labeling them as unequivocally ‘adaptive’ is premature without direct fitness data. An alternative, non‐adaptive interpretation is that these genotype‐specific ‘tight’ environments may simply expose underlying physiological constraints under particular stress combinations. Nevertheless, the observed patterns align with coherent biological narratives rooted in resource allocation theory and life‐history trade‐offs (Stearns 1992; Zera and Harshman 2001). For most genotypes, peak integration occurred under low‐food stress, but diverged along a thermal axis, pointing to distinct strategic priorities. Under combined low‐food and low‐temperature stress (e.g., genotypes A1, A5), the integrated phenotype emphasized tight coordination between key digestive enzymes (e.g., lipase, aminopeptidase) and somatic growth. This architecture prioritizes survival under severe energy deficit: lipids, due to their high energy density, fuel elevated maintenance costs (Olsen et al. 2021), while efficient amino acid utilization supports cellular repair (Broeze et al. 1978; Sokolova et al. 2012), together maximizing energy extraction from scarce intake (Urabe and Watanabe 1992; Yu and Wang 2002; Tian et al. 2023). Since low temperature depresses feeding rates (Loiterton et al. 2004), this coordinated response is crucial to prevent metabolic collapse under the resultant energy deficit. In contrast, under low‐food but moderate‐temperature conditions (e.g., genotypes A6, B), high integration was achieved through coordinated lipid and phosphorus metabolism (Urabe et al. 2018; Tian et al. 2019, 2024). Here, lipids also serve to maintain membrane fluidity (Kumar et al. 2025), while phosphorus, a key structural and functional component, becomes a potential growth‐limiting factor (Elser et al. 2001; Sterner and Elser 2002). Thus, integration under these conditions appears tuned for efficient biomass synthesis when some metabolic activity is permitted but total energy intake remains low.

At a more mechanistic level, genotypes achieving high integration under low‐food/moderate‐temperature conditions converged on the strategic goal of maximizing metabolic return per unit intake, but via distinct enzymatic partnerships. For instance, genotype A6 correlated beta‐glucosidase with lipase (enhancing carbon/lipid harvest; Olsen et al. 2021) and alkaline phosphatase with arginine aminopeptidase (co‐mobilizing limiting phosphorus and nitrogen; Urabe et al. 2018; Tian et al. 2023). Genotype B, however, prioritized different enzyme linkages (e.g., coupling beta‐glucosidase with aminopeptidases for carbohydrate‐nitrogen coordination). Despite different molecular routes, both architectures achieved multi‐substrate coordination, channeling scarce resources into complementary pathways. Thus, rather than a universal response, genotypes employed distinct physiological pathways to achieve high integration under resource scarcity, fine‐tuned to the thermal context. The recurrence of digestive enzyme–body size correlations across environments underscores the fundamental role of energy metabolism–growth coordination. The genotype‐specificity of the “tight” environment highlights how genetic background can shape diverse solutions to similar challenges, contributing to intraspecific variation in phenotypic flexibility (Li et al. 2018; Westerband et al. 2021). This result aligns with the established principle that no single genotype is optimal across all environments, as demonstrated in *Daphnia* by the context‐dependent fitness of metabolic enzyme genotypes under differing resource stoichiometries (Jeyasingh et al. 2009).

Beyond the reorganization of enzyme–enzyme networks, the specific physiological pathways that became most tightly linked to body size also varied among genotypes. This indicates that a functional coupling between nutrient acquisition and growth is a recurring, yet variable, component of the integrated phenotype under energy limitation. The genotype‐specificity of the dominant growth‐integration pathway (e.g., whether linked most strongly to lipid, protein, or combined enzyme activity) suggests that selection has favored diverse physiological solutions to ensure efficient energy allocation toward growth, rather than a single universal molecular coupling. It should be noted that the Nintegration indices were derived from a limited sample size (*n* = 5 per group; see Figure S4 for a power analysis). Nevertheless, the qualitative conclusions are supported by the consistent patterns observed for Aintegration, which is not reliant on significance thresholds, and by convergent results from three complementary matrix‐level approaches, indicating that the context‐dependent reorganization of phenotypic integration is a robust finding.

However, the adaptive value of a specific body size is highly context‐dependent. As shown in Tian et al. (2019, 2024), phenotypic variations in digestive, life‐history, and morphological traits including body size among *D*. cf. *pulex* JPN1 genotypes are driven by food availability and predation. For instance, a larger body size can be advantageous, as it lowers the threshold food level required for positive net growth (Lynch 1977; Gliwicz 1990; Gliwicz and Boavida 1996). Conversely, a smaller body size is favored under high visual predation pressure from planktivorous fish, as it reduces detection risk (Brooks and Dodson 1965; Vanni 1987; Gliwicz and Boavida 1996). Therefore, the observed integration between growth and digestion, whether mediated by lipase, another enzyme, or enzyme combinations, likely functions as a flexible physiological module. It does not constrain body size to a fixed outcome, but instead appears to provide the physiological capacity to achieve a context‐appropriate size, underpinning the evolutionary success of *D*. cf. *pulex* by enabling functional plastic responses to shifting selection pressures.

### 4.3 Environmental Influence Reshapes Phenotypic Architecture Beyond Genetic Constraints

The plasticity of phenotypic integration, as detailed in the previous sections, was found to be largely independent of underlying genetic differences. This decoupling between genome‐wide genetic distance and divergence in phenotypic architecture suggests that the capacity for environmental reorganization of trait relationships is not strongly constrained by the historical genetic divergence among these asexual lineages. While the genetic basis of plasticity and integration has long been theoretically framed (e.g., via G × E and G‐matrices) (Steppan et al. 2002; Josephs 2018), empirical tests of whether neutral genetic divergence predicts their diversification among genotypes remain scarce. A positive correlation between genetic distance and plasticity divergence was observed (*r* = 0.636, *p* = 0.058). Given the wide confidence interval due to the very low statistical power of the test (only 10 pairwise comparisons from five genotypes) and the absence of significant associations for all other traits, this finding does not provide support for a biologically meaningful relationship. Instead, it underscores the need for studies with larger sample sizes to adequately test this hypothesis. Overall, the observed variation in these key phenotypic properties appears not to be strongly constrained by the underlying genetic divergence among the studied genotypes. This decoupling is likely facilitated by the predominantly asexual reproductive mode of this *D*. cf. *pulex* population. In asexual lineages, the recombination of genes is limited (Hill and Robertson 1966; Lande 1984), which could potentially constrain phenotypic evolution. However, the absence of a genetic distance effect indicates that even genetically similar genotypes can exhibit substantial divergence in phenotypic plasticity and integration. This relative independence from strict genetic control may be crucial, as it would allow for greater phenotypic flexibility in response to environmental challenges without being hampered by genetic constraints (Tian et al. 2019).

Our findings indeed demonstrate that environmental factors, rather than genetic distance, are the predominant force shaping the phenotypic architecture of these organisms. The high plasticity and environmental dependency of phenotypic integration mean that a single genotype can express different integration patterns to suit varying conditions. This environmental responsiveness, rather than a fixed genetic program, likely enhances phenotypic flexibility, which is a critical asset for rapid adaptation to heterogeneous or novel environments (Pigliucci 2001; West‐Eberhard 2003; Murren et al. 2005; Richards et al. 2006; Vinton et al. 2022). This mechanism, observed here in *D*. cf. *pulex*, may represent a general strategy for rapid adaptation in other successful asexual invasive lineages. This finding is consistent with the genomic architecture of *D. pulex* , which is characterized by a shared ‘ecoresponsive’ gene repertoire whose dynamic regulation, rather than static genetic variation, underpins its remarkable phenotypic plasticity in the face of environmental change (Colbourne et al. 2011). Beyond *Daphnia*, the context‐dependent integration–plasticity relationship observed here may represent a general strategy among successful clonal invaders. For example, the New Zealand mud snail ( *Potamopyrgus antipodarum* ) and the green alga *Caulerpa racemosa* exhibit high phenotypic plasticity that compensates for limited genetic diversity, enabling colonization of diverse habitats (Geist et al. 2022; Durand et al. 2002). Similarly, invasive plant species such as those in the genus *Turnera* show environment‐dependent integration that correlates with fitness (Damián et al. 2020), suggesting that the capacity for dynamic reorganization of trait architecture may be a common feature of successful invaders across kingdoms. Future comparative studies across asexual and sexual lineages would help determine whether this decoupling of integration from genetic constraints is a general property or specific to certain reproductive modes.

## 5 Conclusion

Our findings contribute to the growing body of evidence challenging the traditional view of phenotypic integration as a static constraint. We demonstrate that in *D*. cf. *pulex*, integration is itself a highly plastic property, dynamically reshaped by genotype‐by‐environment interactions. Crucially, this plasticity of integration does not constrain the magnitude of individual trait responses but rather coordinates them, homogenizing plasticity across integrated traits. This is evidenced by the tight, context‐dependent coupling between digestive enzyme activity and body size. The most notable finding is that this entire reorganizational capacity is largely decoupled from genetic distance, underscoring the predominant role of current environmental drivers over historic genetic constraints.

This study compels a rethinking of the relationship between integration and plasticity. The capacity to maintain core functional modules while flexibly reorganizing trait correlations in response to the environment represents a powerful mechanism for rapid adaptation, paralleling strategies suggested in successful invasive plants where phenotypic integration contributes to ecological versatility (Godoy et al. 2012). We propose that this capacity for environmental reorganization of phenotypic architecture, which appears largely decoupled from strict genetic determinism, could be a key factor facilitating the success of invasive asexual lineages like *D*. cf. *pulex* in heterogeneous environments. Whether this reorganization is directly adaptive or simply permissive requires further investigation, but its existence undoubtedly provides a layer of phenotypic flexibility upon which selection can act. The parallels with other successful clonal invaders suggest that a heavy reliance on integrated phenotypic plasticity may be a common strategy for thriving in heterogeneous environments.

## Author Contributions

**Xiaofei Tian:** conceptualization (equal), formal analysis (equal), investigation (equal), methodology (equal), supervision (equal), writing – original draft (equal), writing – review and editing (equal). **Wenping Feng:** investigation (equal), methodology (equal), writing – original draft (equal), writing – review and editing (equal). **Xiumei Zhang:** formal analysis (equal), supervision (equal).

## Funding

This work was supported by National Natural Science Foundation of China (32301320), Science and Technology Bureau of Zhoushan (2023C41018).

## Conflicts of Interest

The authors declare no conflicts of interest.

## Supporting information


**Table S1:** Genotype id and collected locations of *Daphnia pulex* genotypes used in this study.
**Table S2:** Results of the GLMM analyses examining the effects of integration metrics (Nintegration and Aintegration), environment, and genotype on phenotypic plasticity (PP). (A) Model with Nintegration as the integration predictor; (B) Model with Aintegration as the integration predictor. Coefficient represents estimated regression coefficient for fixed effects. Statistic‐value: for fixed effects (Environment, Nintegration, Aintegration), the Wald *χ* ^2^ statistic from Type III ANOVA is reported; For the random effect (1|Genotype), the variance component of the random intercept is reported, with the corresponding *p*‐value from a likelihood ratio test (LRT). *R* ^2^ represents marginal coefficient of determination (variance explained by fixed effects). AIC represents Akaike Information Criterion.
**Table S3:** Results of the GLMM analyses for the differential plasticity between traits showing the explanatory variables of genotype and maintenance of integration with the coefficients of determination and AIC values. Statistic‐value: for fixed effects (Environment, Nintegration, Aintegration), the Wald *χ* ^2^ statistic from Type III ANOVA is reported; For the random effect (1|Genotype), the variance component of the random intercept is reported, with the corresponding *p*‐value from a likelihood ratio test (LRT). *R* ^2^ represents marginal coefficient of determination (variance explained by fixed effects). AIC represents Akaike Information Criterion.
**Table S4:** Test of equality of the correlation matrices among *Daphnia* cf. *pulex* genotypes within each environment (A), and among environments within each genotype (B). The *χ* ^*2*^ statistic, degrees of freedom (df) and *p*‐values are shown. *p* < 0.05 in bold. Genotype labels: A1, A3, A5, A6, and B are five clonal lines of *Daphnia* cf. *pulex* (JPN1 lineage). Treatment codes are defined as follows: HL, high food (2.0 mg C L^−1^) × low temperature (16°C); LL, low food (0.2 mg C L^−1^) × low temperature (16°C); HM, high food (2.0 mg C L^−1^) × moderate temperature (20°C); LM, low food (0.2 mg C L^−1^) × moderate temperature (20°C); HH, high food (2.0 mg C L^−1^) × high temperature (24°C); LH, low food (0.2 mg C L^−1^) × high temperature (24°C).
**Table S5:** Magnitude of phenotypic integration estimated by an index of integration (INT) for each genotype in each environment. The largest value within each genotype is shown in bold. Values in the ‘Var’ column represent the integration index (INT) calculated from the variance of eigenvalues.
**Table S6:** Summary of mean absolute pairwise Pearson correlations (|*r*|) among the six traits for each of the 30 genotype–environment combinations. Values represent the mean and standard deviation (SD) of the 15 pairwise |*r*| values within each group (*n* = 5). The overall mean |*r*| across all 30 groups is shown at the bottom of the table.
**Figure S1:** Correlation matrix showing Pearson's pairwise correlation coefficients for traits measured for each genotype in each environment. Genotype and environment labels are shown above each correlation matrix panel. Glu, beta‐glucosidase. Lip, lipase. Phos, alkaline‐phosphatase. Arg, arginine amino‐peptidase. Ala, alanine amino‐peptidase. Bl5, body length at day five. Each panel displays the lower triangle of the pairwise correlation matrix. The values are Pearson correlation coefficients (*r*). Asterisks indicate significance levels (*p < 0.05, **p < 0.01). Sample size for each genotype–environment combination was *n* = 5.
**Figure S2:** Power analysis for Pearson correlation at *n* = 5 (*α* = 0.05, two‐tailed). The solid curve with filled circles shows the relationship between effect size (|*r*|) and statistical power. Dashed red line = 80% power threshold; dotted purple line = 90% power threshold. Highlighted points (blue, orange, green) indicate selected effect sizes and their corresponding power values (|*r*| = 0.5, power = 0.21; |*r*| = 0.7, power = 0.49; |*r*| = 0.9, power = 0.87).
**Figure S3:** Phenotypic plasticity of *Daphnia* cf. *pulex* genotypes fitted against Nintegration, and Aintegration in each of five environments. Nintegration means number of statistically significant correlations a trait has with all other measured traits. Aintegration means average squared Pearson correlation coefficient of a trait with all other measured traits. Treatment codes are defined as follows: HL, high food (2.0 mg C L^−1^) × low temperature (16°C); LL, low food (0.2 mg C L^−1^) × low temperature (16°C); HM, high food (2.0 mg C L^−1^) × moderate temperature (20°C); LM, low food (0.2 mg C L^−1^) × moderate temperature (20°C); HH, high food (2.0 mg C L^−1^) × high temperature (24°C); LH, low food (0.2 mg C L^−1^) × high temperature (24°C). Correlation coefficients (*r*) were calculated from transformed data (log_10_(PP + 1)‐transformed plasticity and integration indices). Scatter points and regression lines are based on raw (untransformed) data at the individual level.
**Figure S4:** Difference in plasticity between each pair of two traits across environments in *Daphnia* cf. *pulex* genotypes. The differences in plasticity shown here are derived from the actual phenotypic values of each trait across environments. Trait × Environment interaction indicates that two given traits show differential plasticity, with nonparallel norms of reaction. A significant random effect of genotype implies that all genotypes respond plastically for the two traits in a different way. Glu, beta‐glucosidase, Lip, lipase, Phos, alkaline‐phosphatase, Arg, arginine amino‐peptidase, Ala, alanine amino‐peptidase, Bl5, body length at day five. Treatment codes are defined as follows: HL, high food (2.0 mg C L^−1^) × low temperature (16°C); LL, low food (0.2 mg C L^−1^) × low temperature (16°C); HM, high food (2.0 mg C L^−1^) × moderate temperature (20°C); LM, low food (0.2 mg C L^−1^) × moderate temperature (20°C); HH, high food (2.0 mg C L^−1^) × high temperature (24°C); LH, low food (0.2 mg C L^−1^) × high temperature (24°C). *p* < 0.05 in bold.

## Data Availability

The trait data used in this study are available on Figshare at https://doi.org/10.6084/m9.figshare.32163372.
