# Supplementary material for: Phenotypic Integration Facilitates Plasticity Through Reorganization of Traits Under Multiple Stress: Evidence From Asexual Water Flea Genotypes
Source: Ecol Evol. 2026 Sep 10;16(9):e74304. doi: 10.1002/ece3.74304 (PMC13562911; doi:10.1002/ece3.74304)
Supplement: Supplementary file 1 [file ECE3-16-e74304-s001.docx]

**Supporting Information**

**Title: Phenotypic integration facilitates plasticity through reorganization of traits under multiple stress: evidence from asexual water flea genotypes**

**Methods**

**Power analysis of pairwise Pearson correlation**

To characterize the overall strength of trait associations within each genotype–environment combination, we calculated the pairwise Pearson correlation coefficients among the six traits for each of the 30 groups (5 genotypes × 2 food levels × 3 temperatures, n = 5 per group). For each group, the absolute values of the 15 pairwise correlation coefficients (|*r*|) were extracted from the correlation matrix, and their mean and standard deviation were computed as group-level summary statistics. This yielded 30 sets of mean |*r*| and SD values, representing the average magnitude of trait integration and its variability across trait pairs within each group. These summary statistics were used to evaluate the potential direction of bias (underestimation vs. overestimation) given the limited sample size.

Across all 30 groups, the mean absolute pairwise correlation among the six traits ranged from 0.36 to 0.72, with an overall mean of 0.48 (SD = 0.12). Group-level mean |*r*| values are summarized in Table S6. The modest magnitude of these correlations suggests that the Nintegration values reported here are unlikely to overestimate trait connectedness. When correlations were statistically significant, they were typically very strong (*r* = 0.8–0.99, mostly >0.9), corresponding to a statistical power of approximately 0.84 at n = 5 (Fig.S1 and S2). Consequently, limited statistical power is more likely to cause underestimation than overestimation of trait connectedness, making Nintegration a conservative estimate. It should be noted that this power analysis addresses the detection of statistically significant correlations and therefore directly informs Nintegration. Aintegration, which averages squared correlation coefficients without a significance threshold, is not subject to the same limitation and provides a complementary, magnitude-based measure of integration. A power analysis for detecting significant correlations at n = 5 is provided in Figure S2.

**Worked example for *Dpg* calculation**

To illustrate the calculation of *Dpg*, consider two traits, A and B, measured for a single genotype across two environments (*x* and *y*). The mean values are 10 and 4 for Trait A in environments *x* and *y*, respectively, and 8 and 6 for Trait B. The absolute change for Trait A is |10 − 4| = 6, and for Trait B is |8 − 6| = 2. The numerator is the absolute difference between these two values: ||10 − 4| − |8 − 6|| = |6 − 2| = 4. The denominator is the sum of the two absolute changes: |10 − 4| + |8 − 6| = 6 + 2 = 8. Thus, *Dpg* = 4 / 8 = 0.5. A *Dpg* value of 0.5 indicates a moderate divergence in plasticity magnitude between the two traits. The metric ranges from 0 (identical plasticity magnitude, i.e., both traits change by the same absolute amount) to 1 (maximally divergent plasticity magnitude, i.e., one trait changes while the other remains constant, or the two traits change in opposite directions with equal magnitude). In this example, Trait A changes by 6 units while Trait B changes by 2 units, yielding a *Dpg* of 0.5, reflecting a substantial but not extreme difference in their plastic responses.

**Table S1.** Genotype id and collected locations of *Daphnia* *pulex* genotypes used in this study.

| Genotype ID | Code | Lakes | Prefecture | Latitude/longitude |
| --- | --- | --- | --- | --- |
| A1 | HO03 | Lake Hataya Ohnuma | Yamagata | N 38.245° / E 140.204° |
| A3 | OS02 | Osawa Tame-ike Pond | Miyagi | N 38.439° / E 140.919° |
| A5 | FU01 | Furuichi Oike Pond | Tottori | N 35.391° / E 133.339° |
| A6 | AR01 | Arigatani-ike Pond | Shizuoka | N 34.769° / E 138.135° |
| B | DA04 | Nagano Prefecture | Nagano | N 36.706° / E 138.145° |

**Table S2.** Results of the GLMM analyses examining the effects of integration metrics (Nintegration and Aintegration), environment, and genotype on phenotypic plasticity (PP). (A) Model with Nintegration as the integration predictor; (B) Model with Aintegration as the integration predictor. Coefficient represents estimated regression coefficient for fixed effects. Statistic-value: for fixed effects (Environment, Nintegration, Aintegration), the Wald χ² statistic from Type III ANOVA is reported; For the random effect (1|Genotype), the variance component of the random intercept is reported, with the corresponding *P*-value from a likelihood ratio test (LRT). R² represents marginal coefficient of determination (variance explained by fixed effects). AIC represents Akaike Information Criterion.

**(A)**

| Explanatory  variable | Coefficient | Statistic value | *P* |
| --- | --- | --- | --- |
| Intercept | 0.078 | 8.49 | **<0.001** |
| Environment | - | 25.75 | **<0.001** |
| Nintegration | 0.044 | 10.16 | **<0.001** |
| (1\|Genotype) | variance = 1.4e-4 **0.017** | | |
| Residual | variance = 1.4e-3 | | |
| R^2^ | 0.503 |  |  |
| AIC | -493.32 |  |  |

**(B)**

| Explanatory  variable | Coefficient | Statistic value | *P* |
| --- | --- | --- | --- |
| Intercept | 0.018 | 3.79 | **0.0003** |
| Environment | - | 6.89 | 0.142 |
| Aintegration | 0.32 | 29.25 | **<0.001** |
| (1\|Genotype) | variance = 8.7e-6 | | 0.403 |
| Residual | variance = 3.5e-4 | |  |
| R^2^ | 0.867 |  |  |
| AIC | -699.25 |  |  |

**Table S3.** Results of the GLMM analyses for the differential plasticity between traits showing the explanatory variables of genotype and maintenance of integration with the coefficients of determination and AIC values. Statistic-value: for fixed effects (Environment, Nintegration, Aintegration), the Wald χ² statistic from Type III ANOVA is reported; For the random effect (1|Genotype), the variance component of the random intercept is reported, with the corresponding *P*-value from a likelihood ratio test (LRT). R² represents marginal coefficient of determination (variance explained by fixed effects). AIC represents Akaike Information Criterion.

| Explanatory  variable | Coefficient | Statistic-value | *P* |
| --- | --- | --- | --- |
| Intercept | 0.592 | 3.79 | **<0.001** |
| Maintenance of integration | -0.099 | 29.25 | **<0.001** |
| (1\|Genotype) | variance = 0.00 | | 1.00 |
| Residual | variance = 0.316 | |  |
| R^2^ | 0.015 |  |  |
| AIC | 622.6 |  |  |

**Table S4.** Test of equality of the correlation matrices among *Daphnia* cf. *pulex* genotypes within each environment (A), and among environments within each genotype (B). The *χ^2^* statistic, degrees of freedom (df) and *P*-values are shown. *P* < 0.05 in bold. Genotype labels: A1, A3, A5, A6, and B are five clonal lines of *Daphnia* cf. *pulex* (JPN1 lineage). Treatment codes are defined as follows: HL, high food (2.0 mg C L⁻¹) × low temperature (16°C); LL, low food (0.2 mg C L⁻¹) × low temperature (16°C); HM, high food (2.0 mg C L⁻¹) × moderate temperature (20°C); LM, low food (0.2 mg C L⁻¹) × moderate temperature (20°C); HH, high food (2.0 mg C L⁻¹) × high temperature (24°C); LH, low food (0.2 mg C L⁻¹) × high temperature (24°C).

(A)

| Environment | Genotype pairs | χ2 | df | *P* |
| --- | --- | --- | --- | --- |
| 2.0 mg C L^-1^-16℃ (HL) | A1 vs A3 | 60.33 | 15 | **<0.001** |
|  | A1 vs A5 | 68.37 | 15 | **<0.001** |
|  | A1 vs A6 | 73.90 | 15 | **<0.001** |
|  | A1 vs B | 72.13 | 15 | **<0.001** |
|  | A3 vs A5 | 78.48 | 15 | **<0.001** |
|  | A3 vs A6 | 78.04 | 15 | **<0.001** |
|  | A3 vs B | 74.59 | 15 | **<0.001** |
|  | A5 vs A6 | 72.69 | 15 | **<0.001** |
|  | A5 vs B | 74.53 | 15 | **<0.001** |
|  | A6 vs B | 68.73 | 15 | **<0.001** |
| 0.2 mg C L^-1^-16℃ (LL) | A1 vs A3 | 76.37 | 15 | **<0.001** |
|  | A1 vs A5 | 62.73 | 15 | **<0.001** |
|  | A1 vs A6 | 73.65 | 15 | **<0.001** |
|  | A1 vs B | 75.51 | 15 | **<0.001** |
|  | A3 vs A5 | 74.68 | 15 | **<0.001** |
|  | A3 vs A6 | 87.16 | 15 | **<0.001** |
|  | A3 vs B | 79.54 | 15 | **<0.001** |
|  | A5 vs A6 | 68.13 | 15 | **<0.001** |
|  | A5 vs B | 57.24 | 15 | **<0.001** |
|  | A6 vs B | 74.27 | 15 | **<0.001** |
| 2.0 mg C L^-1^-20℃ (HM) | A1 vs A3 | 56.27 | 15 | **<0.001** |
|  | A1 vs A5 | 66.38 | 15 | **<0.001** |
|  | A1 vs A6 | 61.14 | 15 | **<0.001** |
|  | A1 vs B | 70.56 | 15 | **<0.001** |
|  | A3 vs A5 | 80.95 | 15 | **<0.001** |
|  | A3 vs A6 | 66.61 | 15 | **<0.001** |
|  | A3 vs B | 67.21 | 15 | **<0.001** |
|  | A5 vs A6 | 68.75 | 15 | **<0.001** |
|  | A5 vs B | 87.39 | 15 | **<0.001** |
|  | A6 vs B | 89.54 | 15 | **<0.001** |
| 0.2 mg C L^-1^-20℃ (LM) | A1 vs A3 | 71.23 | 15 | **<0.001** |
|  | A1 vs A5 | 76.11 | 15 | **<0.001** |
|  | A1 vs A6 | 76.69 | 15 | **<0.001** |
|  | A1 vs B | 78.30 | 15 | **<0.001** |
|  | A3 vs A5 | 66.65 | 15 | **<0.001** |
|  | A3 vs A6 | 74.22 | 15 | **<0.001** |
|  | A3 vs B | 74.53 | 15 | **<0.001** |
|  | A5 vs A6 | 83.63 | 15 | **<0.001** |
|  | A5 vs B | 74.87 | 15 | **<0.001** |
|  | A6 vs B | 74.27 | 15 | **<0.001** |
| 2.0 mg C L^-1^-24℃ (HH) | A1 vs A3 | 62.51 | 15 | **<0.001** |
|  | A1 vs A5 | 72.98 | 15 | **<0.001** |
|  | A1 vs A6 | 69.67 | 15 | **<0.001** |
|  | A1 vs B | 75.83 | 15 | **<0.001** |
|  | A3 vs A5 | 66.25 | 15 | **<0.001** |
|  | A3 vs A6 | 62.34 | 15 | **<0.001** |
|  | A3 vs B | 80.71 | 15 | **<0.001** |
|  | A5 vs A6 | 68.59 | 15 | **<0.001** |
|  | A5 vs B | 70.81 | 15 | **<0.001** |
|  | A6 vs B | 81.14 | 15 | **<0.001** |
| 0.2 mg C L^-1^-24℃ (LH) | A1 vs A3 | 44.72 | 15 | **<0.001** |
|  | A1 vs A5 | 85.07 | 15 | **<0.001** |
|  | A1 vs A6 | 78.43 | 15 | **<0.001** |
|  | A1 vs B | 61.01 | 15 | **<0.001** |
|  | A3 vs A5 | 78.15 | 15 | **<0.001** |
|  | A3 vs A6 | 83.88 | 15 | **<0.001** |
|  | A3 vs B | 83.88 | 15 | **<0.001** |
|  | A5 vs A6 | 67.85 | 15 | **<0.001** |
|  | A5 vs B | 67.07 | 15 | **<0.001** |
|  | A6 vs B | 99.69 | 15 | **<0.001** |

(B)

| Genotype | Environment pairs | χ2 | df | *P* |
| --- | --- | --- | --- | --- |
| A1 | HL vs LL | 80.71 | 15 | **<0.001** |
|  | HL vs HM | 73.07 | 15 | **<0.001** |
|  | HL vs LM | 47.16 | 15 | **<0.001** |
|  | HL vs HH | 82.44 | 15 | **<0.001** |
|  | HL vs LH | 64.27 | 15 | **<0.001** |
|  | LL vs HM | 75.52 | 15 | **<0.001** |
|  | LL vs LM | 86.44 | 15 | **<0.001** |
|  | LL vs HH | 80.51 | 15 | **<0.001** |
|  | LL vs LH | 76.83 | 15 | **<0.001** |
|  | HM vs LM | 114.79 | 15 | **<0.001** |
|  | HM vs HH | 77.57 | 15 | **<0.001** |
|  | HM vs LH | 72.27 | 15 | **<0.001** |
|  | LM vs HH | 60.19 | 15 | **<0.001** |
|  | LM vs LH | 61.49 | 15 | **<0.001** |
|  | HH vs LH | 74.13 | 15 | **<0.001** |
| A3 | HL vs LL | 76.62 | 15 | **<0.001** |
|  | HL vs HM | 67.99 | 15 | **<0.001** |
|  | HL vs LM | 68.75 | 15 | **<0.001** |
|  | HL vs HH | 79.36 | 15 | **<0.001** |
|  | HL vs LH | 73.51 | 15 | **<0.001** |
|  | LL vs HM | 84.73 | 15 | **<0.001** |
|  | LL vs LM | 75.82 | 15 | **<0.001** |
|  | LL vs HH | 72.61 | 15 | **<0.001** |
|  | LL vs LH | 70.25 | 15 | **<0.001** |
|  | HM vs LM | 80.31 | 15 | **<0.001** |
|  | HM vs HH | 82.36 | 15 | **<0.001** |
|  | HM vs LH | 73.11 | 15 | **<0.001** |
|  | LM vs HH | 66.58 | 15 | **<0.001** |
|  | LM vs LH | 64.14 | 15 | **<0.001** |
|  | HH vs LH | 70.44 | 15 | **<0.001** |
| A5 | HL vs LL | 75.39 | 15 | **<0.001** |
|  | HL vs HM | 79.97 | 15 | **<0.001** |
|  | HL vs LM | 56.15 | 15 | **<0.001** |
|  | HL vs HH | 76.49 | 15 | **<0.001** |
|  | HL vs LH | 79.13 | 15 | **<0.001** |
|  | LL vs HM | 78.22 | 15 | **<0.001** |
|  | LL vs LM | 77.92 | 15 | **<0.001** |
|  | LL vs HH | 82.74 | 15 | **<0.001** |
|  | LL vs LH | 77.57 | 15 | **<0.001** |
|  | HM vs LM | 75.94 | 15 | **<0.001** |
|  | HM vs HH | 70.13 | 15 | **<0.001** |
|  | HM vs LH | 80.17 | 15 | **<0.001** |
|  | LM vs HH | 66.35 | 15 | **<0.001** |
|  | LM vs LH | 78.81 | 15 | **<0.001** |
|  | HH vs LH | 77.80 | 15 | **<0.001** |
| A6 | HL vs LL | 78.64 | 15 | **<0.001** |
|  | HL vs HM | 110.58 | 15 | **<0.001** |
|  | HL vs LM | 82.51 | 15 | **<0.001** |
|  | HL vs HH | 79.05 | 15 | **<0.001** |
|  | HL vs LH | 63.62 | 15 | **<0.001** |
|  | LL vs HM | 76.35 | 15 | **<0.001** |
|  | LL vs LM | 70.76 | 15 | **<0.001** |
|  | LL vs HH | 78.84 | 15 | **<0.001** |
|  | LL vs LH | 53.02 | 15 | **<0.001** |
|  | HM vs LM | 101.94 | 15 | **<0.001** |
|  | HM vs HH | 72.78 | 15 | **<0.001** |
|  | HM vs LH | 71.60 | 15 | **<0.001** |
|  | LM vs HH | 71.27 | 15 | **<0.001** |
|  | LM vs LH | 73.05 | 15 | **<0.001** |
|  | HH vs LH | 70.73 | 15 | **<0.001** |
| B | HL vs LL | 68.40 | 15 | **<0.001** |
|  | HL vs HM | 75.46 | 15 | **<0.001** |
|  | HL vs LM | 63.85 | 15 | **<0.001** |
|  | HL vs HH | 78.38 | 15 | **<0.001** |
|  | HL vs LH | 66.52 | 15 | **<0.001** |
|  | LL vs HM | 81.17 | 15 | **<0.001** |
|  | LL vs LM | 82.65 | 15 | **<0.001** |
|  | LL vs HH | 81.48 | 15 | **<0.001** |
|  | LL vs LH | 86.72 | 15 | **<0.001** |
|  | HM vs LM | 89.21 | 15 | **<0.001** |
|  | HM vs HH | 75.32 | 15 | **<0.001** |
|  | HM vs LH | 74.17 | 15 | **<0.001** |
|  | LM vs HH | 82.54 | 15 | **<0.001** |
|  | LM vs LH | 79.09 | 15 | **<0.001** |
|  | HH vs LH | 72.54 | 15 | **<0.001** |

**Table S5.** Magnitude of phenotypic integration estimated by an index of integration (INT) for each genotype in each environment. The largest value within each genotype is shown in bold. Values in the ‘Var’ column represent the integration index (INT) calculated from the variance of eigenvalues.

| Genotype | Environment | | Var |
| --- | --- | --- | --- |
|  | Food concentration  (mg C L^-1^) | Temperature (℃) |  |
| A1 | 2.0 | 16 | 0.97 |
|  | **0.2** | **16** | **2.08** |
|  | 2.0 | 20 | 1.00 |
|  | 0.2 | 20 | 1.97 |
|  | 2.0 | 24 | 1.10 |
|  | 0.2 | 24 | 2.06 |
|  | Mean |  | 1.53 |
| A3 | 2.0 | 16 | 1.08 |
|  | 0.2 | 16 | 1.56 |
|  | 2.0 | 20 | 0.96 |
|  | 0.2 | 20 | 1.83 |
|  | **2.0** | **24** | **2.14** |
|  | 0.2 | 24 | 1.16 |
|  | Mean |  | 1.45 |
| A5 | 2.0 | 16 | 1.74 |
|  | **0.2** | **16** | **1.99** |
|  | 2.0 | 20 | 0.98 |
|  | 0.2 | 20 | 1.35 |
|  | 2.0 | 24 | 1.83 |
|  | 0.2 | 24 | 1.95 |
|  | Mean |  | 1.64 |
| A6 | 2.0 | 16 | 1.34 |
|  | 0.2 | 16 | 1.21 |
|  | 2.0 | 20 | 2.15 |
|  | **0.2** | **20** | **2.45** |
|  | 2.0 | 24 | 1.79 |
|  | 0.2 | 24 | 1.21 |
|  | Mean |  | 1.69 |
| B | 2.0 | 16 | 1.28 |
|  | 0.2 | 16 | 1.58 |
|  | 2.0 | 20 | 1.64 |
|  | **0.2** | **20** | **1.92** |
|  | 2.0 | 24 | 0.83 |
|  | 0.2 | 24 | 1.33 |
|  | Mean |  | 1.43 |

**Table S6.** Summary of mean absolute pairwise Pearson correlations (|r|) among the six traits for each of the 30 genotype–environment combinations. Values represent the mean and standard deviation (SD) of the 15 pairwise |r| values within each group (n = 5). The overall mean |r| across all 30 groups is shown at the bottom of the table.

| Genotype | Environment | | Mean | S.D. |  |
| --- | --- | --- | --- | --- | --- |
|  | Food concentration  (mg C L^-1^) | Temperature (℃) |  |  |  |
|  |  |  |  |  |  |
| A1 | 2 | 16 | 0.37 | 0.07 |  |
|  | 0.2 | 16 | 0.62 | 0.07 |  |
|  | 2 | 20 | 0.37 | 0.04 |  |
|  | 0.2 | 20 | 0.58 | 0.13 |  |
|  | 2 | 24 | 0.37 | 0.13 |  |
|  | 0.2 | 24 | 0.60 | 0.11 |  |
|  | Mean |  | 0.48 |  |  |
| A3 | 2 | 16 | 0.40 | 0.09 |  |
|  | 0.2 | 16 | 0.48 | 0.17 |  |
|  | 2 | 20 | 0.39 | 0.05 |  |
|  | 0.2 | 20 | 0.63 | 0.09 |  |
|  | 2 | 24 | 0.56 | 0.21 |  |
|  | 0.2 | 24 | 0.41 | 0.10 |  |
|  | Mean |  | 0.48 |  |  |
| A5 | 2 | 16 | 0.48 | 0.11 |  |
|  | 0.2 | 16 | 0.57 | 0.13 |  |
|  | 2 | 20 | 0.37 | 0.09 |  |
|  | 0.2 | 20 | 0.45 | 0.15 |  |
|  | 2 | 24 | 0.44 | 0.10 |  |
|  | 0.2 | 24 | 0.72 | 0.13 |  |
|  | Mean |  | 0.50 |  |  |
| A6 | 2 | 16 | 0.42 | 0.15 |  |
|  | 0.2 | 16 | 0.41 | 0.13 |  |
|  | 2 | 20 | 0.45 | 0.15 |  |
|  | 0.2 | 20 | 0.65 | 0.13 |  |
|  | 2 | 24 | 0.55 | 0.09 |  |
|  | 0.2 | 24 | 0.45 | 0.07 |  |
|  | Mean |  | 0.49 |  |  |
| B | 2 | 16 | 0.40 | 0.19 |  |
|  | 0.2 | 16 | 0.51 | 0.11 |  |
|  | 2 | 20 | 0.48 | 0.06 |  |
|  | 0.2 | 20 | 0.53 | 0.20 |  |
|  | 2 | 24 | 0.36 | 0.06 |  |
|  | 0.2 | 24 | 0.40 | 0.11 |  |
|  | Mean |  | 0.45 |  |  |

**Fig. S1.** Correlation matrix showing Pearson’s pairwise correlation coefficients for traits measured for each genotype in each environment. Genotype and environment labels are shown above each correlation matrix panel. Glu, beta-glucosidase. Lip, lipase. Phos, alkaline-phosphatase. Arg, arginine amino-peptidase. Ala, alanine amino-peptidase. Bl5, body length at day five. Each panel displays the lower triangle of the pairwise correlation matrix. The values are Pearson correlation coefficients (*r*). Asterisks indicate significance levels (**P* < 0.05, ** *P* < 0.01). Sample size for each genotype–environment combination was n = 5.

**Fig. S2.** Power analysis for Pearson correlation at n = 5 (α = 0.05, two-tailed). The solid curve with filled circles shows the relationship between effect size (|*r*|) and statistical power. Dashed red line = 80% power threshold; dotted purple line = 90% power threshold. Highlighted points (blue, orange, green) indicate selected effect sizes and their corresponding power values (|*r*| = 0.5, power = 0.21; |*r*| = 0.7, power = 0.49; |*r*| = 0.9, power = 0.87).

**Fig. S3.** Phenotypic plasticity of *Daphnia* cf. *pulex* genotypes fitted against Nintegration, and Aintegration in each of five environments. Nintegration means number of statistically significant correlations a trait has with all other measured traits. Aintegration means average squared Pearson correlation coefficient of a trait with all other measured traits. Treatment codes are defined as follows: HL, high food (2.0 mg C L⁻¹) × low temperature (16°C); LL, low food (0.2 mg C L⁻¹) × low temperature (16°C); HM, high food (2.0 mg C L⁻¹) × moderate temperature (20°C); LM, low food (0.2 mg C L⁻¹) × moderate temperature (20°C); HH, high food (2.0 mg C L⁻¹) × high temperature (24°C); LH, low food (0.2 mg C L⁻¹) × high temperature (24°C). Correlation coefficients (*r*) were calculated from transformed data (log₁₀(PP+1)-transformed plasticity and integration indices). Scatter points and regression lines are based on raw (untransformed) data at the individual level.

**Fig. S4.** Difference in plasticity between each pair of two traits across environments in *Daphnia* cf. *pulex* genotypes. The differences in plasticity shown here are derived from the actual phenotypic values of each trait across environments. Trait (T) × Environment (E) interaction indicates that two given traits show differential plasticity, with nonparallel norms of reaction. A significant random effect of genotype (G) implies that all genotypes respond plastically for the two traits in a different way. Glu, beta-glucosidase; Lip, lipase; Phos, alkaline-phosphatase; Arg, arginine amino-peptidase; Ala, alanine amino-peptidase; Bl5, body length at day five. Treatment codes are defined as follows: HL, high food (2.0 mg C L⁻¹) × low temperature (16°C); LL, low food (0.2 mg C L⁻¹) × low temperature (16°C); HM, high food (2.0 mg C L⁻¹) × moderate temperature (20°C); LM, low food (0.2 mg C L⁻¹) × moderate temperature (20°C); HH, high food (2.0 mg C L⁻¹) × high temperature (24°C); LH, low food (0.2 mg C L⁻¹) × high temperature (24°C). *P* < 0.05 in bold.
